# German Version of the mHealth App Usability Questionnaire in a Cohort of Patients With Cancer: Translation and Validation Study

**DOI:** 10.2196/51090

**Published:** 2023-11-01

**Authors:** Preetha Moorthy, Lina Weinert, Bendix Christian Harms, Carolin Anders, Fabian Siegel

**Affiliations:** 1 Department of Biomedical Informatics, Center for Preventive Medicine and Digital Health, Medical Faculty Mannheim Heidelberg University Mannheim Germany; 2 Institute of Medical Informatics Heidelberg University Hospital Heidelberg Germany; 3 Section for Translational Health Economics, Department for Conservative Dentistry Heidelberg University Hospital Heidelberg Germany

**Keywords:** usability, mobile health, mHealth app, questionnaire validation, questionnaire translation, mHealth App Usability Questionnaire, MAUQ, mobile phone

## Abstract

**Background:**

Good usability is important for the adoption and continued use of mobile health (mHealth) apps. In particular, high usability can support intuitive use by patients, which improves compliance and increases the app’s effectiveness. However, many usability studies do not use adequate tools to measure perceived usability. The mHealth App Usability Questionnaire (MAUQ) was developed specifically for end users in a medical context. MAUQ is a relatively new but increasingly used questionnaire to evaluate mHealth apps, but it is not yet available in German.

**Objective:**

This study aims to translate MAUQ into German and determine its internal consistency, reliability, and construct validity.

**Methods:**

This validation study was conducted as part of a usability evaluation project for an mHealth app used as a therapy support tool during breast cancer chemotherapy. MAUQ was translated into German through a rigorous forward-backward translation process, ensuring semantic and conceptual equivalence. Patient responses to MAUQ and System Usability Scale (SUS) were analyzed for validation. Descriptive analysis was performed for the MAUQ subscales and SUS standard scores. Significance tests and correlation coefficients assessed the relationship between the SUS and MAUQ results, confirming construct validity. Internal consistency was assessed for item reliability and consistency in measuring the target construct. Free-text questions assessed translation comprehensibility, with responses analyzed descriptively and qualitatively using content analysis.

**Results:**

In this study, 133 participants responded to the questionnaire, and the validation analysis showed substantially positive correlations between the overall MAUQ score and its subscales: ease of use (*r*=0.56), interface and satisfaction (*r*=0.75), and usefulness (*r*=0.83). These findings support the construct validity of MAUQ and emphasize the importance of these subscales in assessing the usability of the Enable app. The correlation coefficients ranging from 0.39 to 0.68 for the items further validate the questionnaire by aligning with the overall score and capturing the intended concept. The high internal consistency reliability of MAUQ (Cronbach α=.81) and its subscales further enhances the instrument’s robustness in accurately evaluating the usability of mHealth apps.

**Conclusions:**

We successfully validated the German translation of the MAUQ for stand-alone apps using a standardized approach in a cohort of patients with breast cancer. In our validation study, MAUQ exhibited strong internal consistency reliability (Cronbach α=.81) across its subscales, indicating reliable and consistent measurement. Furthermore, a significant positive correlation (*P*<.001) was found between the subscales and the overall score, supporting their consistent measurement of the intended construct. Therefore, MAUQ can be considered a reliable instrument for assessing the usability of mHealth apps among German-speaking adults. The availability of the German version of MAUQ will help other researchers in conducting usability studies of mHealth apps in German-speaking cohorts and allow for international comparability of their results.

## Introduction

### Background

The use of digital technology in both routine health care and research continues to rise. The increasing adoption of fitness and medical apps has reached a global market size of US $43.5 billion in 2022. This increment is projected to experience a compound annual growth rate of 11.6% from 2023 to 2030 [1]. As defined by the World Health Organization (WHO) Global Observatory for eHealth, mobile health (mHealth) encompasses medical and public health practices supported by mobile devices, such as mobile phones, patient monitoring devices, PDAs, and other wireless devices by health care professionals or patients [2]. According to Morse et al [3], mHealth apps refer to software integrated into smartphones with the aim of enhancing health outcomes, health research, and health care services. The continuous increase of reporting, data collection, telemedicine, and emergency medical care using mHealth apps draws attention to the acceptance and user experience of the targeted users. Knowledge about these factors could assist physicians and health care providers in choosing the right mHealth apps for their patients. However, many research studies do not adequately evaluate mHealth interventions nor provide sufficient evidence about the health impact [2-4]. To facilitate such studies, it is essential to establish and reach a consensus on standardized indicators and metrics for monitoring and evaluating purposes. Questionnaires are one of the well-established methods that are widely used in research, clinical trials, and health care settings to gather data and measure various constructs. One of the oldest usability measurement scales, the System Usability Scale (SUS) [5], was developed in 1986 to assess the usability of electronic office systems. Nowadays, SUS is widely used and considered a highly reliable tool for evaluating software, websites, or mobile apps.

When questionnaires need to be used in different cultural and linguistic contexts, it is essential to ensure their equivalence across languages. The identification of the construct to be evaluated using the questionnaire is critical, as it defines the scope of interest and determines the type of measurements that will be obtained. The limited availability of standardized usability questionnaires in languages other than English poses a potential challenge when assessing system usability and user experience among non–English-speaking populations. Only a few usability-related evaluation questionnaires are currently available in the German language such as International Organization for Standardization (ISO) Norm 9241/110 [6], AttrakDiff [7], Scale for Measuring Perceived Website Usability [8], Software Usability Measurement Inventory [9], User Experience Questionnaire [10], and Mobile Application Rating Scale [11].

Compared with the abovementioned and established usability evaluation questionnaires, the mHealth App Usability Questionnaire (MAUQ) [12] is a relatively new questionnaire in this area. Therefore, it has been used less than other established questionnaires such as SUS [13]. In contrast to the Mobile Application Rating Scale, which is also used to evaluate mHealth apps, MAUQ was developed specifically and initially for the target group of end users in a medical context. However, owing to its specialization for mHealth apps, MAUQ is becoming increasingly popular internationally and has been translated into various languages [14-18]. In the German context, some studies have used self-translated, nonvalidated versions of MAUQ. This shows the need for a validated, translated version of MAUQ for use in German-speaking populations [19-24].

### Objectives

This study aimed to achieve a linguistically and conceptually equivalent version of the stand-alone version of the MAUQ instrument in German for the target population.

## Methods

### Overview of the Questionnaire

MAUQ, developed and validated by Zhou et al [12], assesses the usability of mHealth apps among patients and health care providers. The stand-alone version of MAUQ was used in this study. MAUQ consists of 18 items distributed across 3 dimensions: ease of use (5 items), interface and satisfaction (7 items), and usefulness (6 items). These dimensions capture various aspects related to the usability of the assessed mHealth apps. Using a 7-point Likert scale ranging from 1 (strongly agree) to 7 (strongly disagree), participants rated each item in the questionnaire. Usability was determined by calculating the total and average scores of all statements, where high average scores indicate a high level of usability. The results showed that strong internal consistency was observed in the overall MAUQ for stand-alone apps used by patients (Cronbach α=.914). The respective subscales of MAUQ also exhibited strong internal consistency reliability, as evidenced by Cronbach α coefficients of ease of use (Cronbach α=.847), interface and satisfaction (Cronbach α=.908), and usefulness (Cronbach α=.717).

### Participants

Participants were recruited as part of the project, Multicenter Digital Recording of Patient Satisfaction, Quality of Life, and Patient-Reported Adverse Events in Breast Carcinoma in Neoadjuvant, Adjuvant, Follow-Up, and Palliative Care, in short, ENABLE [25], a randomized controlled trial (RCT) that aimed to improve patients’ adherence to breast cancer therapy. We followed the recommendations proposed in previous studies [26-28], particularly when conducting the exploratory factor analysis, and used the sample-to-item ratio to determine an appropriate sample size based on the number of items in the study. In accordance with the suggested criterion of maintaining a sample-to-item ratio of no less than 5:1, a preliminary calculation led us to determine an appropriate sample size of 90 before initiating the validation study. It is noteworthy that MAUQ comprises 18 items, and as per the established recommendation, each item necessitates responses from 5 participants [26-28].

Participants were screened to meet the following inclusion criteria: diagnosis of invasive or metastatic breast cancer and planning neoadjuvant, adjuvant, or palliative therapy; minimum age of 18 years; German language skills; and possession of a tablet or smartphone with internet access. Participants were eligible for this study once they were enrolled in the ENABLE study.

### Procedure

The aim was to use a standardized approach to translate a usability questionnaire and to ensure that the instrument is equally natural and acceptable and functions effectively across different cultural contexts. An established approach to accomplish this objective is by using forward translations and backward translations. This is a widely used approach to evaluate the comprehensibility of a source text and to identify any errors or uncertainties that may require attention or rectification while finalizing the text to perform translation followed by backward translation [29-31].

### Study Context

This validation study was conducted in the context of the ENABLE project, which investigated the use of an mHealth app as a therapy support tool for patients undergoing chemotherapy for breast cancer. The studied mHealth app, called Enable app, provides the opportunity to conduct reactive patient-reported outcome assessments, such as screening for adverse events, health-related quality of life, and patient satisfaction. In addition, the app offers patients information about their therapy, medication, and common adverse events. The app also visualizes the patient’s therapy progress using a progress bar and includes information about upcoming appointments. Both the clinical effectiveness and the usability of this newly developed app were addressed in the ENABLE study, a randomized clinical trial conducted at 3 university hospitals in Germany. The usability of the Enable app was measured through a combination of qualitative measures (semistructured interviews and eye tracking) and quantitative measures (MAUQ and SUS).

### Ethical Considerations

The study was conducted in accordance with the Declaration of Helsinki and was approved by the ethics committee of the Heidelberg University Hospital (S-685/2020). Confidentiality and anonymity were ensured throughout the entire study. Study participants provided written informed consent.

### Translation Process

#### Overview

**Figure 1:**
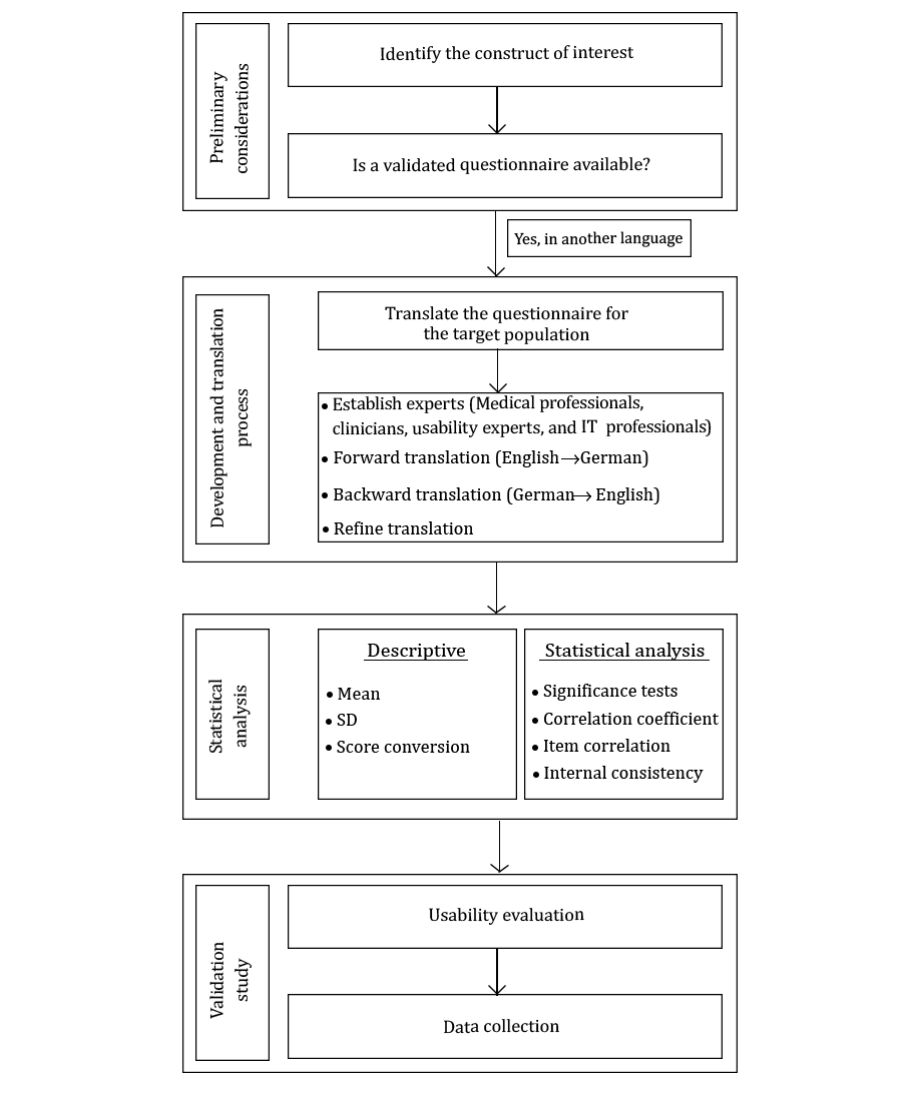
Depiction of the translation process for the purpose of this study.

MAUQ was translated from English to German with the help of translators certified by the ISO 17100:2015 norm. We adapted the WHO guidelines for the translation process (Figure 1): (1) forward translation, (2) expert panel, (3) backward translation, and (4) refine translation [32]. To ensure accuracy and consistency in the translation process from the source to the target language, 2 independent and certified translators performed the translation [33-35]. The different versions of the translation and the final German translation of MAUQ are included in Multimedia Appendices 1 and 2, respectively.

#### Forward Translation

During this phase, an experienced and certified translator, proficient in both the source and target languages, carefully translated the questionnaire from the source language, English, to the target language, German. In this way, the linguistic nuances, idiomatic expressions, and cultural references captured can be compared with the original meaning and intent of the questions. This step ensured that the translated questionnaire was understandable to the target population and maintained the integrity of the original instrument.

#### Expert Panel

For this phase, a review panel was created, involving skilled experts from different domains, namely, clinicians, medical informaticians, and usability experts. The panel consisted of members who were bilingual and proficient in both the English and the German language. The experts were asked to review all the translations and identify and resolve the concepts of translation that are inadequate. In case of discrepancies between the forward translation and the original, experts could address specific words or expressions in the translated questionnaire and provide alternative suggestions.

#### Backward Translation

Following the forward translation, a second independent translator who is also ISO certified, experienced, fluent in the target language, and proficient in the source language performed the backward translation. This translator independently translated the questionnaire back to the source language. The purpose of the backward translation was to compare the translated version with the original questionnaire, allowing for an assessment of linguistic accuracy and potential inconsistencies.

#### Refine Translation

The expert panel thoroughly reviewed the backward-translated version and compared it with the original questionnaire and the forward translation to identify any differences or contrarieties. Irregularities found during the comparison were discussed and reconciled through collaboration among the expert panel and the project stakeholders. This step involved a careful examination of the wording, structure, and semantics of the items. The goal was to ensure that the final translated version maintains semantic and conceptual equivalence with the original questionnaire.

### Data Collection

Data collection for this study was conducted from May 2021 to October 2022. MAUQ was administered at 2 different time points: once at 4 weeks after the individual study started and again after 20 weeks. All patients enrolled in the ENABLE project at the Department of Gynecology and Obstetrics, Heidelberg University Hospital, received a printed version of MAUQ and SUS (10 wk after the individual study started) via postal mail. The original, translated surveys were complemented with additional questions developed by the authors (eg, regarding sociodemographic data, use of other mHealth apps, history of smartphone ownership, and understandability of the newly translated MAUQ). Patients were asked to return the completed questionnaires via postal mail.

Survey data were collected and managed using REDCap (Research Electronic Data Capture; Vanderbilt University) electronic data capture tools [36] hosted at the Heidelberg University Hospital. All data were exported from REDCap to R statistical software (version 4.0.4; R Foundation for Statistical Computing). All data were checked for plausibility and analyzed by the study team members.

### Statistical Analysis

#### Descriptive Analysis

An initial descriptive analysis was conducted to examine participant demographics and assess the performance of MAUQ and SUS, by calculating means and SDs for individual items and the overall questionnaire. In this study, MAUQ included 18 items, which were categorized into 3 subscales: ease of use (items 1-5), interface and satisfaction (items 6-12), and usefulness (items 13-18). We calculated the scores for each subscale and the total score for MAUQ, with all scores ranging from 0 to 100 [12]. In addition, we used a standard score conversion procedure for the SUS questionnaire to convert participants’ responses into scores ranging from 0 to 100 [37].

#### Significance Tests

We used the significance tests to assess the statistical significance of differences or relationships within SUS, MAUQ, and MAUQ’s subscales. These tests help determine whether the observed findings reflect actual effects or relationships within the population rather than random chance. In addition, the discriminative ability assesses the questionnaire’s capacity to differentiate between varying levels of attributes, capturing subtle distinctions in responses for meaningful comparisons and conclusions [38,39]. As such, identifying significant differences between the highest and lowest quintile means is crucial.

#### Correlation Coefficient and Internal Consistency

The correlation coefficients were computed to assess the relationships among MAUQ SUS, and the subscales of MAUQ to determine the construct validity [37,40,41]. These coefficients quantify the degree of association among these scores, with values close to 1 or –1 indicating a strong correlation. In addition, Kendall rank correlation coefficient was used to examine the relationship between the individual MAUQ items and the overall score. Furthermore, internal consistency was assessed to reflect on the interrelatedness of MAUQ items, indicating questionnaire reliability and validity. Cronbach α measured the internal consistency, with values between 0.7 and 0.8 considered acceptable and values around 0.9 considered excellent. High values signify strong item consistency and enhanced questionnaire validity [42-44].

#### Comprehensibility

At the end of the translated MAUQ survey, additional questions were added regarding the understandability of the translation (“Was the text in the above questionnaire easy to understand? Yes/No. If not, why not? Do you have any further feedback on the above questionnaire you would like to share with us?”). These questions were added to ensure that participants felt invited to share thoughts or feedback about how to improve the understandability of the translated survey. Any feedback entered in these free-text fields was exported from REDCap to Microsoft Excel and analyzed descriptively by LW and CA. In addition, content analysis was performed to understand the rates of occurrence of the identified themes in the free-text fields.

## Results

### Overview

After the exclusion of incomplete questionnaires, 133 questionnaires were included in the analysis. For the purpose of this validation study, completed MAUQs from both data collection time points were considered. Overall, 75.9% (101/133) of the participants returned the SUS questionnaire. Tables 1 and 2 show the characteristics of the participants.

**Table 1:** Sociodemographic data of participants (N=133).

Characteristics		Participants, n (%)
**Sex**		
	Female	133 (100)
**Age group (y)**		
	<30	2 (1.5)
	30-40	16 (12)
	41-50	48 (36.1)
	51-60	42 (31.6)
	61-70	18 (13.5)
	71-80	7 (5.3)
**Education**		
	Academic degree	48 (36.1)
	High school education	17 (12.8)
	Lower or intermediate secondary school	62 (46.6)
	Prefer not to say	6 (4.5)
**Employment**		
	Employed	79 (59.4)
	Unemployed	25 (18.8)
	Studying or vocational training	1 (0.8)
	Retired	18 (13.5)
	Prefer not to say	10 (7.5)

**Table 2:** Additional participant characteristics regarding smartphone and app use.

Characteristics		Values
Length of smartphone ownership (y; N=133), mean (SD)		11 (4.96)
**Use of other mHealth^a^ apps (N=133), n (%)**		
	Yes	44 (33.1)
	No	83 (62.4)
	Prefer not to say	6 (4.5)
**Use of wearables (N=133), n (%)**		
	Yes	50 (37.6)
	No	77 (57.9)
	Prefer not to say	6 (4.5)
**Frequency of Enable app use (n=101), n (%)**		
	Daily or several days a week	48 (47.5)
	Once a week	46 (45.5)
	Once a month or less	6 (5.9)
	Prefer not to say	1 (0.9)

^a^mHealth: mobile health.

### Descriptive Statistics

In this study, descriptive statistics were evaluated for SUS and MAUQ to assess the usability of the Enable app. The participants’ high level of perceived usability, as indicated by the SUS score with a mean of 88.3 (SD 9.9), noticeably outperformed the average score of 68. Similarly, MAUQ yielded a mean score of 85.89 (SD 11.45), further affirming the favorable perception of usability. As questionnaire results were comparable at the 2 different data collection time points and to achieve a high validation sample, it was decided to combine the results of the 2 time points for the purpose of this validation study.

The normal distribution of the data was assessed using the Shapiro-Wilk test. The normalization analysis using the Shapiro-Wilk test indicated that none of the items in the questionnaire followed a normal distribution, as evidenced by the small *P* values (*P*<.001) obtained, indicating a low likelihood of these items conforming to normality (Table 3). In addition, a visual examination of the histograms further confirms this deviation from normality, as the Shapiro-Wilk test may sometimes overestimate the departure from a normal distribution. Furthermore, these findings suggest that nonparametric statistical tests should be used for further analysis. The results of the normalization analysis are shown in Table 4.

**Table 3:** Means and SDs of the subscales of the mHealth App Usability Questionnaire.

Subscale	Score, mean (SD)
Ease of use	6.445 (0.749)
Interface and satisfaction	6.223 (0.847)
Usefulness	5.405 (1.144)

**Table 4:** Results of the normalization analysis using the Shapiro-Wilk test.

Item	Shapiro-Wilk test *P* value	Score, mean (SD)
1	<.001	6.704 (0.773)
2	<.001	6.76 (0.723)
3	<.001	6.6 (0.823)
4	<.001	5.912 (1.426)
5	<.001	6.248 (1.175)
6	<.001	6.24 (1.103)
7	<.001	6.232 (1.086)
8	<.001	5.784 (1.543)
9	<.001	5.992 (1.292)
10	<.001	6.544 (0.875)
11	<.001	6.456 (1.096)
12	<.001	6.312 (1.066)
13	<.001	5.968 (1.373)
14	<.001	5.472 (1.532)
15	<.001	5.408 (1.498)
16	<.001	5.32 (1.511)
17	<.001	4.848 (1.778)
18	<.001	5.416 (1.52)

### Significance Tests

The Wilcoxon rank sum test was conducted to assess the significance of differences in the scores of MAUQ and SUS. Significant differences were found in the mean scores between the overall scores of SUS and MAUQ. In addition, significant differences were also observed in the mean scores for the ease of use and usefulness subscales of MAUQ, whereas no significant difference was found for the interface and satisfaction subscales. We evaluated the discriminative ability of the questionnaire items; the Wilcoxon rank sum test was used to compare the highest-scoring and lowest-scoring quintiles. The analysis revealed significant differences in means for all items, indicating their ability to discriminate between the different levels of usability.

### Correlation Coefficient

This study investigated the association between the subscales and overall scores of MAUQ using Kendall rank correlation coefficient. The results revealed a moderate positive correlation between the ease of use subscale and the overall scores (*r*=0.56; *P*<.001), indicating that as the perceived ease of use increased, so did the overall scores. Moreover, a strong positive correlation was observed between the interface and satisfaction subscale and the overall scores (*r*=0.75; *P*<.001), suggesting that high levels of satisfaction with the interface were associated with high overall scores. In addition, a high positive correlation was found between the usefulness subscale and the overall scores (*r*=0.83; *P*<.001), indicating that the great perceived usefulness of the app was linked to high overall scores. These findings support the construct validity of MAUQ and highlight the importance of these subscales in assessing the usability of the Enable app.

The total item correlation was also computed for the German version of MAUQ (G-MAUQ) using Kendall rank correlation coefficient, with a predefined threshold value of 0.4. Our results showed that the correlation coefficients ranged from 0.39 to 0.68. These values indicate moderate to strong associations between the items and the overall score of G-MAUQ. This suggests that the items in the questionnaire collectively contribute to the measurement of the construct assessed by the questionnaire. Correlation coefficients of the overall score and subscales of MAUQ are shown in Table 5.

**Table 5:** Correlation coefficients of the overall score and subscales of the mHealth App Usability Questionnaire.

Subscale	Ease of use	Interface and satisfaction	Usefulness	Overall score
Ease of use	1	0.4176	0.4496	0.5649
Interface and satisfaction	0.4176	1	0.5879	0.7475
Usefulness	0.4496	0.5879	1	0.8295
Overall score	0.5649	0.7475	0.8295	1

### Internal Consistency

The intersubscale internal consistency reliability of MAUQ was evaluated using Cronbach α coefficient (Table 6). The obtained Cronbach α value of .81 suggests satisfactory internal consistency reliability among the subscales. This indicates that the items within MAUQ consistently measure related aspects of usability. Furthermore, the internal consistency reliability within each subscale was assessed using Cronbach α coefficient. The ease of use subscale demonstrated a value of .79, indicating good internal consistency. Similarly, the interface and satisfaction subscale exhibited a value of .85, and the usefulness subscale showed a value of .84, both indicating strong internal consistency within their respective subscales. These results suggest that the items within each subscale are measuring a similar construct consistently.

**Table 6:** Internal consistency reliability of the mHealth App Usability Questionnaire (MAUQ).

Subscales of MAUQ	Cronbach α
Ease of use	.7857
Interface and satisfaction	.8497
Usefulness	.8375
Overall score	.8102

### Comprehensibility

In the translated MAUQ, 95.5% (127/133) of the participants answered the additional yes or no question about understandability, and 95.3% (121/127) replied that the survey was easy to understand. In total, 51 comments were obtained in the free-text fields. Many comments (23/25, 92%) referred to the ENABLE study itself or technical problems experienced in the Enable app. Thus, these comments were excluded for the purpose of this analysis. In the following step, the 55% (28/51) remaining comments were analyzed and categorized into 6 groups. Most comments (14/28, 50%) comprised short, positive statements, such as “good,” “questionnaire was quick and easy,” and “everything is comprehensible.” Another common group of comments (5/28, 18%) covered the wish to add more free-text space in the questionnaire to enable participants to make suggestions for improvement. Some comments (3/28, 11%) described having difficulties with understanding the Likert scale or the impression that questions were very similar and hard to differentiate from each other (3/28, 11%). Overall, 7% (2/28) of the participants noted that they experienced difficulty with understanding the questionnaire owing to their low command of the German language, and 4% (1/28) of the participants described that question 8 included a term that they could not understand (“The app adequately acknowledged...”).

## Discussion

### Principal Findings

We conducted a translation and validation study of G-MAUQ in a cohort of 133 German-speaking patients with breast cancer. The determination of an appropriate sample size for questionnaire validation lacks universally prescribed guidelines. However, it is generally recommended to use a large sample size to achieve a high respondent-to-question ratio to enhance the statistical robustness of the analysis. In our study, we adhered to the recommendation of maintaining a ratio of at least 5 participants per statement (MAUQ items=18) to ensure an adequate sample size for the questionnaire validation [26-28]. Importantly, our achieved ratio surpassed this threshold, meeting the recommended criterion for a sufficient sample size.

Data were collected in the context of an RCT studying the use of an mHealth app as a support tool during the course of chemotherapy. In our validation study, we observed a positive correlation between the subscales and the overall score of G-MAUQ. However, our findings suggest that the discrepancy in scores compared with the original validation study [12] could be attributed to the differences in participant characteristics. The previous study recruited participants primarily from the University of Pittsburgh, with a limitation being that approximately one-third of their participants were students. In contrast, our validation study included actual patients with breast cancer who were enrolled in the RCT of the ENABLE project. The contrasting health statuses of healthy participants in the previous study and patients with chronic illness in our study could potentially influence the obtained scores.

On the basis of our results from the statistical analysis, we observed that the correlations support the validity of G-MAUQ in capturing the intended concept and provide evidence of the items’ alignment with the overall scoring of the instrument. Correspondingly, the high internal consistency reliability observed across the subscales of G-MAUQ strengthens the confidence in its ability to accurately measure usability in the context of mHealth apps. These findings support the reliability and validity of G-MAUQ as a tool for assessing the different dimensions of usability in this population. This is consistent with the findings from the Chinese and Malay version of MAUQ [15,16].

During the translation process, it was observed that certain words in German did not align perfectly with the original English word and its intended meaning. In addition, in German, some words can have multiple meanings depending on the context. This highlighted the influence of cultural factors on translation, similar to a previous study [16]. To ensure a precise and accurate understanding of the German words within the usability context, modifications were made to the wording. These adjustments aimed to clarify the purpose and meaning of the questionnaire items. For example, consider item 9 within the questionnaire, which incorporates the phrase, “social settings.” The term, “social,” in this context presents 2 potential translations in German: “soziales Umfeld,” signifying the social environment encompassing friends, family, and even unfamiliar individuals within one’s social sphere, and “gesellschaftliches Umfeld,” which refers to the societal environment, including factors such as a person’s upbringing, education, and care. These divergent interpretations of the term, “social,” signify a notable variance in how it is perceived and understood. After careful deliberation, we opted for “gesellschatliches Umfeld” (societal environment) as it conveys a more comprehensive and contextually fitting interpretation, closely aligning with the intended meaning of “social settings” in item 9.

Similarly, in item 11, which states, “I would use the app again,” the word “use” in the German language carries various connotations and interpretations. Initially, we translated “use” as “verwenden,” implying reuse, such as using the app in a different context or situation. However, this translation did not precisely capture the intended sense of the English phrase with its specific context. Therefore, following a thorough examination and expert review by native speakers, we chose to use the literal translation of the term, “use” (nutzen), signifying the act of using the app once more in its original context. This highlighted the influence of cultural factors on translation, similar to the studies by Zhou et al [16]. Furthermore, the results of our qualitative assessment indicated that the translated MAUQ was easily comprehensible based on the modifications implemented.

Regarding the feedback about questionnaire understandability and language, overall, participants perceived the understandability to be adequate, and the questionnaire was easy to complete for most (121/127, 95.3%). Thus, we do not plan to make any further changes to the translation. However, participants provided 51 additional comments in the free-text fields, giving feedback about the overall study, study team members, and the app. In these comments, 18% (5/28) voiced the desire to have more space for individual feedback about the app in the usability questionnaire. Hence, we recommend future users of G-MAUQ to also provide a free-text field when administering the survey, prompting the participants to provide valuable, individual, additional feedback.

Although it may be tempting for researchers to develop new, study-specific questionnaires for their studies, using a validated questionnaire holds several advantages. First, developing a new questionnaire requires a lot of resources and takes time [45]. Second, owing to the extensive validation processes, the validity of established questionnaires is high, which makes the results more trustworthy. Another aspect is that results derived from validated questionnaires can be more easily compared with results from other studies on similar topics. This also applies to validated translations of existing questionnaires. Using these validated translations ensures the comparability of research findings across different cultural contexts and languages [46]. To the best of the authors’ knowledge, there are 3 other validated translations of MAUQ available [15-18]. The availability of these validated translations will be helpful in conducting population-specific and methodologically sound studies of mHealth usability.

### Strengths and Limitations

Our study followed a structured approach of WHO’s Back-Translation Guidelines [32] to validate a self-developed German translation of MAUQ. Data were collected within a large research project, and a sufficient number of participants completed the survey, allowing for sound statistical analysis. In addition, individual feedback about the understandability and wording of the questionnaire was collected. This allowed us to assess the quality of the translation from the perspective of laypersons. To demonstrate the external validity of our findings, we also recommend that future studies should investigate whether the translated questionnaire can be used effectively in the context of other mHealth apps.

In an effort to include feedback from as many participants as possible, even those with low technical capabilities or who were experiencing difficulties with using the Enable app, we decided to collect data through mailed questionnaires. This approach was both time-consuming for the study team and could have introduced mistakes owing to the necessary manual data entry. Another effort that was made to increase the study sample was the combination of questionnaires from 2 different time points. This was only possible because the results from the 2 different time points did not differ in a noteworthy manner. However, this combination could have introduced a potential bias in our validation.

Owing to the study being conducted in the context of a large breast cancer trial, our study sample included only female participants. Although this is an important limitation to note, we do not consider the sex of the participants to play an influential role in the validity and understandability of the questionnaire. This was shown by previous studies, which concluded that there are no significant differences between female and male study participants regarding the perceived usability of a system [47-49].

However, 36.1% (48/133) of the participants in our sample held an academic degree. This is above average compared with the share of academics in the German population overall (24%) [50]. In addition, 1.5% (2/133) of the participants stated that they had difficulties in understanding the questionnaire owing to their low command of the German language. Future research projects should make additional efforts to include participants from these traditionally underrepresented groups (low educational backgrounds and nonnative speakers) in their samples.

### Conclusions

We successfully validated G-MAUQ using a standardized approach in a cohort of 133 patients with breast cancer. Similar to the original version, G-MAUQ revealed good reliability and validity in this study. Our validation study demonstrated robust and satisfactory internal consistency reliability among the subscales of MAUQ, with a Cronbach α coefficient of .81, indicating strong and satisfactory reliability. In addition, we observed a significant positive correlation between the subscales and the overall score of MAUQ. These results indicate a high degree of internal consistency and support the construct validity of MAUQ. Hence, it can be used as a reliable tool to evaluate the usability of mHealth apps among German-speaking adults. The availability of G-MAUQ will help other researchers in conducting usability studies of mHealth apps in German-speaking cohorts and allow for international comparability of their results. Further research is recommended to study the validity of the translated questionnaire in other user groups and in other contexts for mHealth apps.

## Appendix

Multimedia Appendix 1 Translations of the mHealth App Usability Questionnaire in German and English.

Multimedia Appendix 2 German version of the mHealth App Usability Questionnaire.

## Abbreviations

G-MAUQ: German version of mHealth App Usability Questionnaire
ISO: International Organization for Standardization
MAUQ: mHealth App Usability Questionnaire
mHealth: mobile health
RCT: randomized controlled trial
REDCap: Research Electronic Data Capture
SUS: System Usability Scale
WHO: World Health Organization
